# Pharmacological Inhibition of HMGB1 Prevents Muscle Wasting

**DOI:** 10.3389/fphar.2021.731386

**Published:** 2021-11-18

**Authors:** Lu Li, Huiquan Liu, Weili Tao, Su Wen, Xiaofen Fu, Shiying Yu

**Affiliations:** ^1^ Cancer Center, Tongji Hospital, Tongji Medical College, Huazhong University of Science and Technology, Wuhan, China; ^2^ Department of Radiation Oncology, The First Affiliated Hospital of Anhui Medical University, Hefei, China

**Keywords:** HMGB1, cancer cachexia, skeletal muscle, NF-κB, TLR4

## Abstract

**Background:** Cachexia is a multifactorial disorder characterized by weight loss and muscle wasting, making up for about 20% of cancer-related death. However, there are no effective drugs to combat cachexia at present.

**Methods:** In this study, the effect of CT26 exosomes on C2C12 myotubes was observed. We compared serum HMGB1 level in cachexia and non-cachexia colon cancer patients. We further explored HMGB1 expression level in CT26 exosome. We added recombinant HMGB1 to C2C12 myotubes to observe the effects of HMGB1 on C2C12 myotubes and detected the expression level of the muscle atrophy-related proteins. Then, we used the HMGB1 inhibitor glycyrrhizin to reverse the effects of HMGB1 on C2C12 myotubes. Finally, HMGB1 inhibitor glycyrrhizin was utilized to relieve cachexia in CT26 cachexia mouse model.

**Results:** Exosomes containing HMGB1 led to muscle atrophy with significantly decreased myotube diameter and increased expression of muscle atrophy-related proteins Atrogin1 and MuRF1. Further, we detected that HMGB1 induced the muscle atrophy mainly *via* TLR4/NF-κB pathway. Administration of the HMGB1 inhibitor glycyrrhizin could relieve muscle wasting *in vitro* and attenuate the progression of cachexia *in vivo*.

**Conclusion:** These findings demonstrate the cachectic role of HMGB1, whether it is soluble form of HMGB1 or secreted from tumor cells as part of exosomes. HMGB1 inhibitor glycyrrhizin might be a promising drug in colon cancer cachexia.

## Introduction

Cancer cachexia is a multifactorial symptom with the feature of weight loss and muscle wasting, which is caused by a combination of various reasons, including reduced food intake, metabolic changes, excess catabolism, and inflammation (Biswas and Acharyya, 2020). It is a systemic metabolic disorder syndrome that cannot be completely reversed by nutritional support. The incidence of cachexia is about 80% in pancreatic cancer and gastric cancer, 50% in lung cancer and colorectal cancer, and 40% in breast cancer (Dewys et al., 1980; Teunissen et al., 2007; Argilés et al., 2014). Muscle wasting is the main feature of cancer cachexia patients. It is related to the declined quality of life, decreased immunity, increased susceptibility to infection, decreased tolerance to treatment, increased postoperative mortality and shortened survival time (AuthorAnonymous, 2018).

Exosomes (EVs), deriving from endosomal compartment of numerous cells, are released outside the cells *via* plasma membrane fusion and participant in cell communication (Théry, 2011). EVs contain lots of cargos, including proteins, mRNA, lncRNA, miRNA, nucleic acids, taking a pivotal role in various physiological and pathological process (Kalra et al., 2016). Emerging evidence has confirmed that tumor-derived exosomes could induce cancer cachexia. EVs containing HSP70 and HSP90 have been reported to trigger cancer cachexia (Zhang et al., 2017a). Esophageal cancer derived exosomes containing P4HB could induce muscle wasting through PHGDH/Bcl-2/caspase-3 pathway (Gao et al., 2021).

Systemic inflammation is one of the main causes of cachexia muscle wasting. Pro-inflammatory cytokines such as IL-6, TNF α, and IL-1β promote the transcriptional activation of atrophy-related genes Atrogin1 and MuRF1, which play a major role in the occurrence and development of muscle wasting. Activation of inflammatory pathways such as NF-κB signaling pathway could cause muscle wasting both in cachexia mouse models and muscles of cancer cachexia patients (Guttridge et al., 2000; Zhou et al., 2003; Rhoads et al., 2010; Lu et al., 2021). Inflammatory factors can activate the NF-κB signaling pathway, and the activation of the NF-κB signaling pathway can reversely enhance inflammation by promoting the transcription of inflammatory factors (Camargo et al., 2015; Lu et al., 2021). This is a vicious circle. Extracellular high mobility group box protein B1 (HMGB1) is an important mediator involved in the pathogenesis of many acute and chronic inflammation. Serum HMGB1 of colon cancer patients was significantly higher than that of healthy subjects (Lee et al., 2012). Furthermore, toll-like receptor 4 (TLR4) and receptor for advanced glycation end‐products are two dominant receptors of HMGB1 (Andersson et al., 2018). Those two receptors and their downstream signaling pathways (NF-κB signaling pathway and MAPK signaling pathway) have been reported to facilitate muscle catabolism (Zhang et al., 2017b; Chiappalupi et al., 2020; Ono et al., 2020). Meanwhile, TLR4/NF-κB signaling pathway has been reported to mediate muscle wasting in septic mice and patients of chronic kidney disease (Verzola et al., 2017; Hahn et al., 2020). Thus, we infer that HMGB1 might be related to muscle wasting. However, there are few studies on the relationship between HMGB1 and cachexia at present.

In this article, we explored the effects of EVs containing HMGB1 on C2C12 myotubes. Then, we used the HMGB1 inhibitor glycyrrhizin to confirm the cachectic role and tried to find out the possible pathways. *In vivo* study confirmed the cachexia effects of HMGB1 and anti-cachexia effects of glycyrrhizin on CT26 tumor-bearing mice.

## Materials and Methods

### Cell Culture and Reagents

Mouse CT26 colon cancer cells (CT26 cells) and mouse myoblasts (C2C12 cells) were purchased from Shanghai Institutes for Biological Sciences, Chinese Academy of Sciences. CT26 cells and C2C12 were cultured in Dulbecco’s modified Eagle’s medium (DMEM) supplemented with 10% fetal bovine serum (FBS), 1% penicillin G-streptomycin (Gibco, MA, United States) in 37°C incubators with 5% CO2. When C2C12 myoblasts reached 90%, the proliferation medium was discarded and replaced with DMEM medium containing 2% horse serum (Gibco, MA, United States). The differentiation medium was replaced every day. Glycyrrhizin was obtained from SelleckChem (Huston TX, United States) and dissolved in dimethyl sulfoxide (DMSO). TAK-242, BAY 11-7082, PD169316 were purchased from MedChem Express (NJ, United States).

When the cell density of CT26 reaches 80%, discard the complete medium and wash with sterile PBS gently for at least 3 times. Then continue to incubate for 48 h with serum-free DMEM. The CT26 cell supernatant was collected and centrifuged at 1,200 rpm for 10 min to remove the cell debris. Filter the centrifuged CT26 supernatant through a 0.2 μm sterile syringe filter and the medium was used as a cachectic factor. CT26 conditioned medium (CCM) were prepared by CT26 supernatant and DMEM at a ratio of 1:1 with 2% horse serum.

### Extraction and Quantification of Exosomes

The CT26 supernatant was centrifuged at 500 × *g* for 15 min at 4°C, 3,000 × *g* for 15 min, and 12,000 × *g* for 30 min to remove tumor cells and fragments. The exosomes were precipitated by an ultracentrifuge (Beckman, Brea, CA, United States) at 120,000 × *g* for 90 min. The supernatant was collected as exosome-depleted CCM. The pallet was resuspended in PBS and the exosomes were purified by centrifugation at 120,000 × *g* for 90 min. The resulting exosome pellet was resuspended in PBS for cell experiments. The exosome pellet obtained after ultracentrifugation was lysed with radioimmunoprecipitation assay (RIPA) for 30 min, and then centrifuged at 12,000 g for 30 min at 4°C. The supernatants containing total proteins were transferred to a new centrifuge tube, and proteins in exosomes were quantified by bicinchoninic acid (BCA) assay (Beyotime Biotechnology). Murine serum exosomes were obtained by ultracentrifugation using the above method after dilution with PBS at a ratio of 1:1.

### Transmission Electron Microscopy to Observe the Morphology of Exosomes

The exosomes derived from CT26 were fixed with 1% glutaraldehyde in PBS (pH 7.4) and then washed. Take 30 μl of exosome suspension on the formvar/carbon coated grid, and then negatively stain with 3% (W/V) aqueous phosphotungstic acid for 90 s, and observe with a transmission electron microscope (Hitachi H-7000FA).

### Exosome Uptake Experiment

The exosomes from CT26 were labeled with 2 μM PKH26 (Sigma-Aldrich) for 4 min. Complete medium was used to neutralize with PKH26, then the excess dye was removed by ultracentrifugation for an hour. The precipitation was re-suspended in PBS and incubated with C2C12 myotube cells for 8 h. The cells were then counterstained with 2-(4-amidinophenyl)-1H-indole-6-carboxamidine (DAPI) and observed by fluorescence microscope (Leica, Germany).

### Particle Size Analysis

We analyzed the fraction of exosomes on a ZETAVIEW instrument (Particle Metrix, Germany). Use nanoparticle tracking analysis software to count and analyze EV particles.

### Patients Cohort and Data Acquisition

The cross-sectional study collected patients with advanced colon cancer in Tongji hospital (Wuhan, Hubei province, China) from May 2020 to September 2020. Ethical approval was granted by the Committee on the Ethics of Tongji hospital. The inclusion criteria: the patients were pathologically diagnosed as advanced colon cancer, no history of other malignancies, clear consciousness, more than 18 years old, blood samples available, available abdominal CT scan within 1 month, no previous diabetes, no thyroid disease and no other factors that may cause weight loss. Cancer cachexia was diagnosed according to the international consensus (Fearon et al., 2011).

### Elisa

Human serum HMGB1 were quantified by the HMGB1 ELISA Kit (Elabscience, Wuhan, China). After anesthesia, mouse blood samples were obtained through the retro-orbital sinus. Serums were collected by centrifugation at 2,500 rpm at 4°C for 15 min for ELISA test. IL-6 (Bio-swamp, Wuhan, China), TNF-α (Bio-swamp, Wuhan, China), HMGB1 (Elabscience, Wuhan, China) were quantitatively detected according to the instructions.

### Immunofluorescence Staining

After treating C2C12 myotubes in a 24-well plate with different concentrations of HMGB1 for 72 h, they were washed 3 times with PBS, fixed with 4% paraformaldehyde for 20 min, and permeabilized with 0.5% Triton X-100 at room temperature for 30 min. The primary antibody MyHC antibody was incubated overnight at 4°C, and then incubated with Alexa Fluor 488 goat anti-mouse IgG antibody (1:500; Invitrogen) at room temperature for 1 h, and the cells were stained with DAPI. Images of myotubes were taken by a fluorescence microscope. The diameters of more than 100 myotubes in at least 10 fields were measured by ImageJ software (Zhang et al., 2017a).

### Western Blotting

Proteins were extracted in RIPA lysis buffer with 1% phenylmethylsulphonyl fluoride (PMSF) and 1% phosphorylase inhibitor cocktail (Servicebio, Wuhan, China) for 30 min at 4°C. Proteins were quantified by BCA assay. Each sample of 30 μg protein was electrophoresed on 10% SDS-PAGE gel. Then, electro-transfer proteins to polyvinylidene difluoride (PVDF) membranes. Membrane were incubated with primary antibodies overnight at 4°C after being blocked in 5% bovine serum albumin at room temperature for 1 h. Wash the membranes in Tris-buffered saline with 0.1% Tween-20 (TBST) for 3 times. The membranes were incubated with the corresponding secondary antibody conjugated with horseradish peroxidase (dilution 1:10,000, Aspen Biotechnology, Wuhan, China). Finally, membranes were detected using ECL solution (Servicebio Technology, Wuhan, China) following TBST washed for 3 times. The antibodies used were listed as follows: NF-κB p65 (p65; dilution 1:1,000; cat# 8242; Cell Signaling Technology, Danvers, MA, United States), phosphorylated NF-κB p65 (P-p65; dilution 1:1,000; cat# 3033, Ser536; Cell Signaling Technology), IκBα (cat# 4814; Cell Signaling Technology, Danvers, MA, United States), phosphorylated IκBα (cat#2859; Cell Signaling Technology, Danvers, MA, United States), MuRF1 (dilution 1:1,000; cat# 55,456–1-AP; Proteintech Technology, Wuhan, China), Atrogin1 (dilution 1:1,000; cat# ab168372; Abcam, Cambridge, MA, United States), α-tubulin (dilution 1:5,000; cat# 11224-1-AP; Proteintech, Wuhan, China), Anti-myosin heavy chain (cat#MAB4470, R&D Systems, Minneapolis, MN). The HRP-conjugated anti-mouse and anti-rabbit antibodies were obtained from Invitrogen. Replicates of western blotting were shown in Supplementary Figure.

### Lentiviral Transduction

For HMGB1 knockdown vector, the sequences used were: ATG​CAG​CTT​ATA​CGA​AGA​TAA. The sequences of scramble shRNA (shCON) were: TTC​TCC​GAA​CGT​GTC​ACG​T. When CT26 cells reached 50% confluence, we replaced the complete medium with a serum-free medium containing 5 μg/ml polybrene and lentiviruses (GV493, GeneChem, Shanghai, China) at a multiplicity of infection of 50. Then, change to complete medium after 12 h. The empty vector lentivirus transduction was performed in the same way. When GFP was detected under the fluorescence microscope, we added puromycin to the complete medium at a final concentration of 3 μg/ml for 1 week to select stable transfected cells.

### Animal Studies

All experimental protocols were approved by the Institutional Animal Care and Use Committee at Tongji Medical College, Huazhong University of Science and Technology and were in accordance with the National Institutes of Health Guide for the Care and Use of Laboratory Animals. Six weeks old healthy male BALB/c mice were raised in a regular 12 h light-dark cycle and specific pathogen free environment.

To explore the role of HMGB1 *in vivo*, mice were randomly divided into five groups (n = 8 per group): control group, CT26 group, shCON group, shHMGB1 group, shHMGB1+EVs group. The control group was normal mice without CT26 injection, and the CT26 group mice were injected subcutaneously with untreated CT26. The mice in the shCON group were injected with CT26 cells transduced with empty lentiviruses. The mice in the shHMGB1 group were injected with HMGB1-lowexpressed CT26 cells. The mice in shHMGB1+EVs group were injected with HMGB1-lowexpressed CT26 cells and 20 μg CT26 exosomes were injected three times a week. The remaining mice were injected with 100ul of PBS. The weight of mice and the size of tumors were recorded every 3 days.

For glycyrrhizin treatment in CT26 cachexia model, mice were randomly divided into four groups (n = 8 per group): healthy mice without tumor, CT26 tumor-bearing mice without glycyrrhizin treatment, CT26 tumor-bearing mice with glycyrrhizin (5 mg/kg/day) treatment, CT26 tumor-bearing mice with glycyrrhizin (20 mg/kg/day) treatment. Tumor-bearing mice were injected subcutaneously with CT26 cells (1 × 10^6^) into the right flank and healthy mice were injected with equal volume of PBS. Treatment started 7 days after tumor implantation when the tumors were palpable. Mice in the control group were injected with equivalent drug vehicle. Mice in glycyrrhizin group were treated with glycyrrhizin every day for 2 weeks (intraperitoneal injection, i. p.). The body weights of the mice, food intake and tumor volumes were measured every 3 days. Two weeks after treatment, mice were euthanized. Tumors, gastrocnemius muscles and epididymal adipose tissues were removed, weighed, and quickly frozen in liquid nitrogen for subsequent analysis.

### HE Staining

The mice gastrocnemius were fixed in 4% paraformaldehyde and embedded in paraffin. Then, paraffin-embedded tissues were sectioned transversely and stained with hematoxylin and eosin solutions. Morphological images were acquired using an optical microscope. Cross-sectional areas of myofibers were quantified by ImageJ software.

### Statistical Analysis

The results were presented as the mean ± SD. Student t-test or one-way ANOVA was used to analyze the differences. A value of *p* < 0.05 was considered statistically significant. All experiments were performed at least three times. The control groups of independent experiments were normalized to one without showing variations (actual variations were within the normal range) under applicable conditions (Zhang et al., 2011; Ding et al., 2017; Sin et al., 2021). * represented *p* value < 0.05, ** represented *p* value < 0.01, *** represented *p* value < 0.001.

## Results

### Isolation and Identification of CT26-Derived Exosomes

**FIGURE 1:**
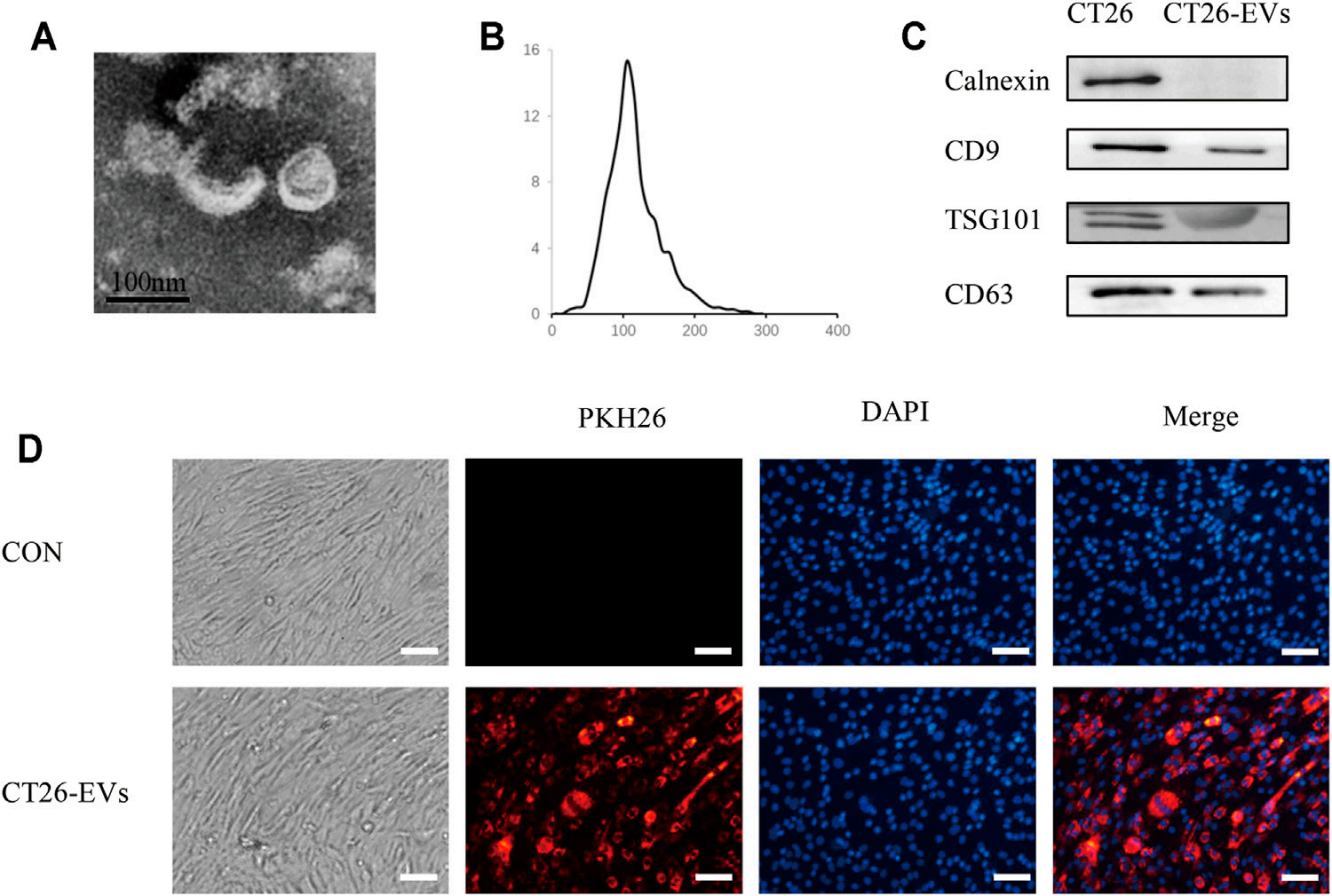
Isolation and identification of CT26-derived exosomes (CT26-EVs). **(A)** Transmission electron microscopy of isolated exosomes from CT26 cells. Scale bar = 100 nm. **(B)** The concentration and size distribution were determined by nanoparticle tracking analysis. **(C)** Western blot of exosomal marker in exosomes derived from CT26 cells. **(D)** PKH26 treated exosomes up-taken by C2C12 myotubes. C2C12 myotubes were incubated with PKH26-labelled CT26-EVs **(red)**. C2C12 myotubes were stained with DAPI **(blue)**. Scale bar = 100 μm.

To study the effect of CT26-derived exosomes on C2C12 myotubes, we extracted the CT26-derived exosomes through ultracentrifugation. The characteristics of the exosomes were obtained through transmission electron microscopy, nanoparticle tracking analysis (NTA) and western blot. We found double-layered vesicles, with the diameters of most particles were around 110 nm (Figures 1A,B). WB analysis showed the known exosomal marker proteins CD9, TSG101, CD63 on CT26-derived exosomes, while the negative protein Calnexin was undetectable (Figure 1C). Moreover, we incubated C2C12 myotubes with PKH26-labeled exosomes and observed that these exosomes could be taken up by C2C12 myotubes (Figure 1D).

### CT26-Derived Exosomes can Induce Muscle Wasting in C2C12 Myotubes

**FIGURE 2:**
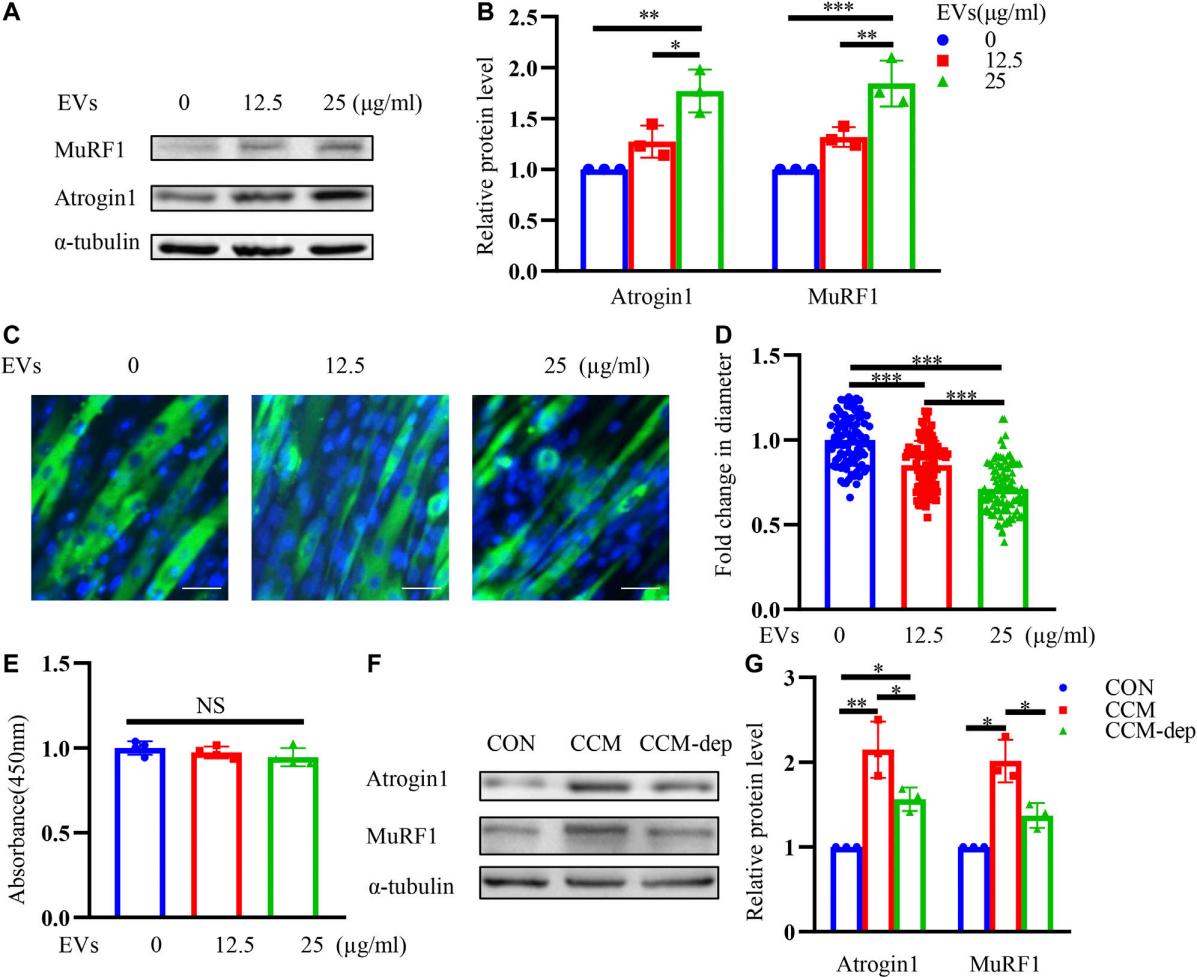
CT26-derived exosomes induce muscle wasting in C2C12 myotubes. **(A)** Total extracts of C2C12 myotubes treated with different concentrations of CT26-EVs were used for western blotting analysis with antibodies against with Atrogin1, MuRF1 and α-tubulin. **(B)** Densitometric quantification of the Atrogin1 and MuRF1 signal relative to the α-tubulin signal. **(C)** Representative immunofluorescence images for C2C12 myotubes treated with different concentrations of CT26-EVs. Scale bar = 50 μm. **(D)** The relative fiber widths of C2C12 myotubes treated with different concentrations of CT26-EVs. **(E)** CCK8 assay for C2C12 myotubes treated with different concentrations of CT26-EVs. **(F)** Total extracts of C2C12 myotubes treated with CT26-EVs and exosome-depleted conditioned medium were used for western blotting analysis with Atrogin1, MuRF1 and α-tubulin. **(G)** Densitometric quantification of the Atrogin1 and MuRF1 signal relative to the α-tubulin signal.

Incubating C2C12 myotubes with different concentrations of CT26-EVs for 24 h, we detected higher expression of muscle atrophy-related proteins Atrogin1 and MuRF1, indicating that a certain level of CT26-EVs could induce muscle wasting in C2C12 myotubes (Figures 2A,B). Furthermore, we conducted immunofluorescence staining assay to observe the changes of myotube diameter (Figure 2C) and calculated myotubes diameters of different groups (Figure 2D). We found that CT26-derived exosomes could significantly induce muscle atrophy by western blot and immunofluorescence staining assay. At the same time, EVs could not induce apoptosis in myotubes inferring from the CCK-8 assay (Figure 2E). CCM was reported to induce muscle wasting (Zhang et al., 2017c). To further verify the importance of EVs in myotube atrophy, we collected exosome-depleted CCM to compare the effects with normal CCM. As shown in Figures 2F,G, muscle atrophy-related proteins Atrogin1 and MuRF1 decreased when EVs were depleted. Therefore, EVs were cachexia factors in CCM-mediated C2C12 myotube atrophy.

### HMGB1 is Upregulated in CT26-Derived Exosomes and HMGB1 can Induce Muscle Wasting *in Vitro*

**FIGURE 3:**
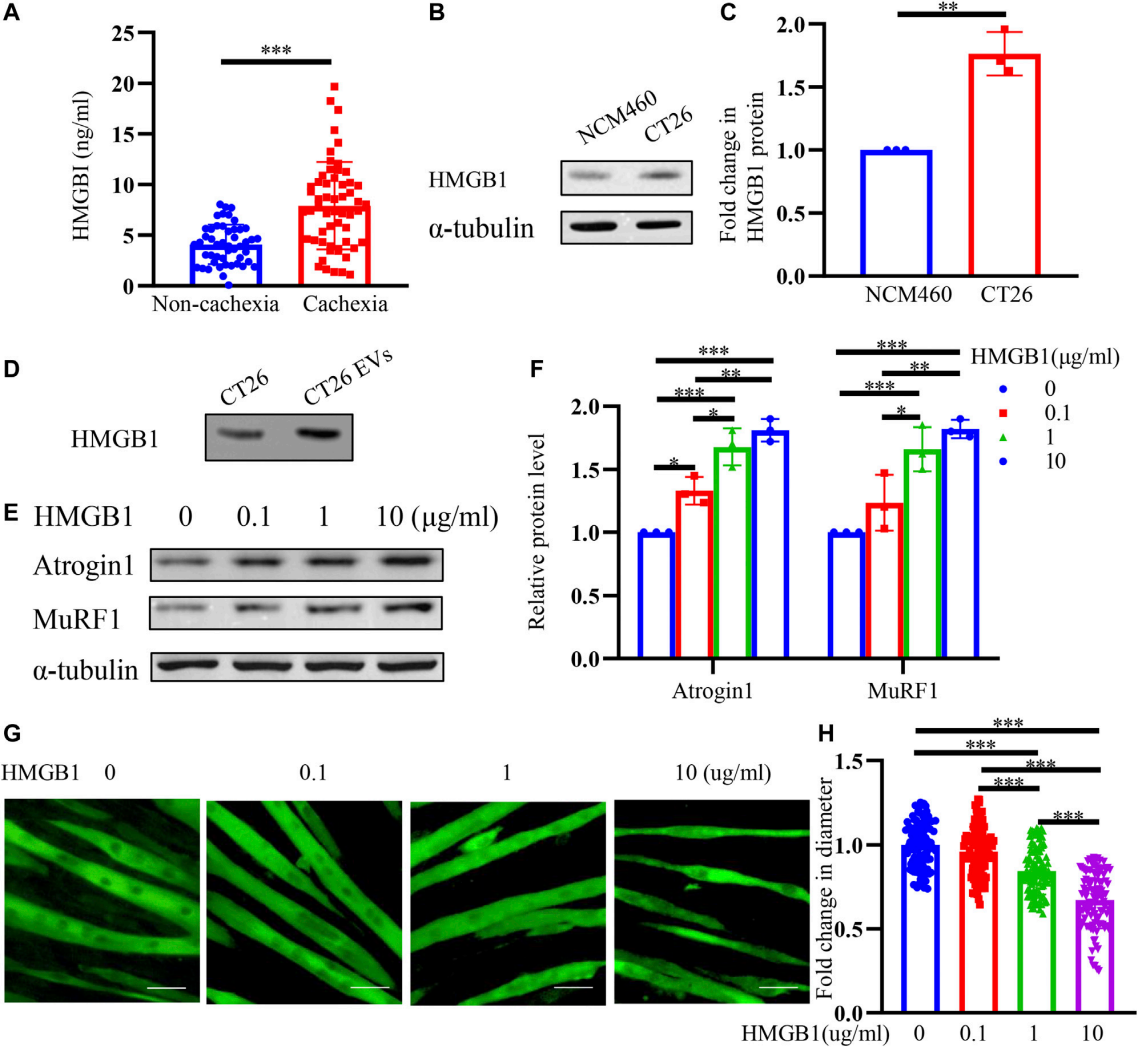
HMGB1 is upregulated in CT26-derived exosomes and HMGB1 induces muscle wasting *in vitro*. **(A)** The serum HMGB1 in colon cancer cachexia patients was higher than non-cachexia patients. **(B)** HMGB1 expression was higher in CT26 cells than normal colonic epithelium (NCM460 cells). **(C)** Densitometric quantification of the HMGB1 signal relative to the α-tubulin signal. **(D)** CT26-EVs contained high level of HMGB1 protein. **(E)** Total extracts of C2C12 myotubes treated with different concentrations of recombinant HMGB1 were used for western blotting analysis with antibodies against with Atrogin1, MuRF1 and α-tubulin. **(F)** Densitometric quantification of the Atrogin1 and MuRF1 signal relative to the α-tubulin signal. **(G)** Representative immunofluorescence images for C2C12 myotubes treated with different concentrations of recombinant HMGB1. Scale bar = 50 μm. **(H)** The relative fiber widths of C2C12 myotubes treated with different concentrations of recombinant HMGB1.

HMGB1 has been reported to participate in inflammation and cancer (Vijayakumar et al., 2019; Cheng et al., 2020; Xue et al., 2021). Studies have shown that HMGB1 is elevated in both sera and tumor tissues of colon cancer patients (Cheng et al., 2020). HMGB1 promotes the proliferation, invasion, and metastasis of colon cancer cells (Zhang et al., 2015; Zhu et al., 2015; Chandrasekaran et al., 2016). We infer that HMGB1 may take a role in muscle wasting. We collected serum of colon cancer patients and found that HMGB1 was remarkably increased in cachexia patients than non-cachexia (Figure 3A). We also detected significantly higher HMGB1 expression in CT26 cancer cells than the normal colonic epithelium (NCM460 cells) (Figures 3B,C). And we also found a high expression of HMGB1 in CT26-EVs (Figure 3D). Therefore, we evaluated the effects of HMGB1 on C2C12 myotubes *in vitro*. Different concentrations of recombinant HMGB1 were added to C2C12 myotubes. The expression of atrophy-related genes Atrogin1 and MuRF1 were elevated (Figures 3E,F). Combined with the morphology of the myotubes (Figure 3G) and myotube diameter (Figure 3H), recombinant HMGB1 could induce myotube atrophy in a dose-dependent manner (0.1–10 μg/ml).

### HMGB1 Induces Muscle Wasting Through TLR4/NF-κB Signaling Pathway

**FIGURE 4:**
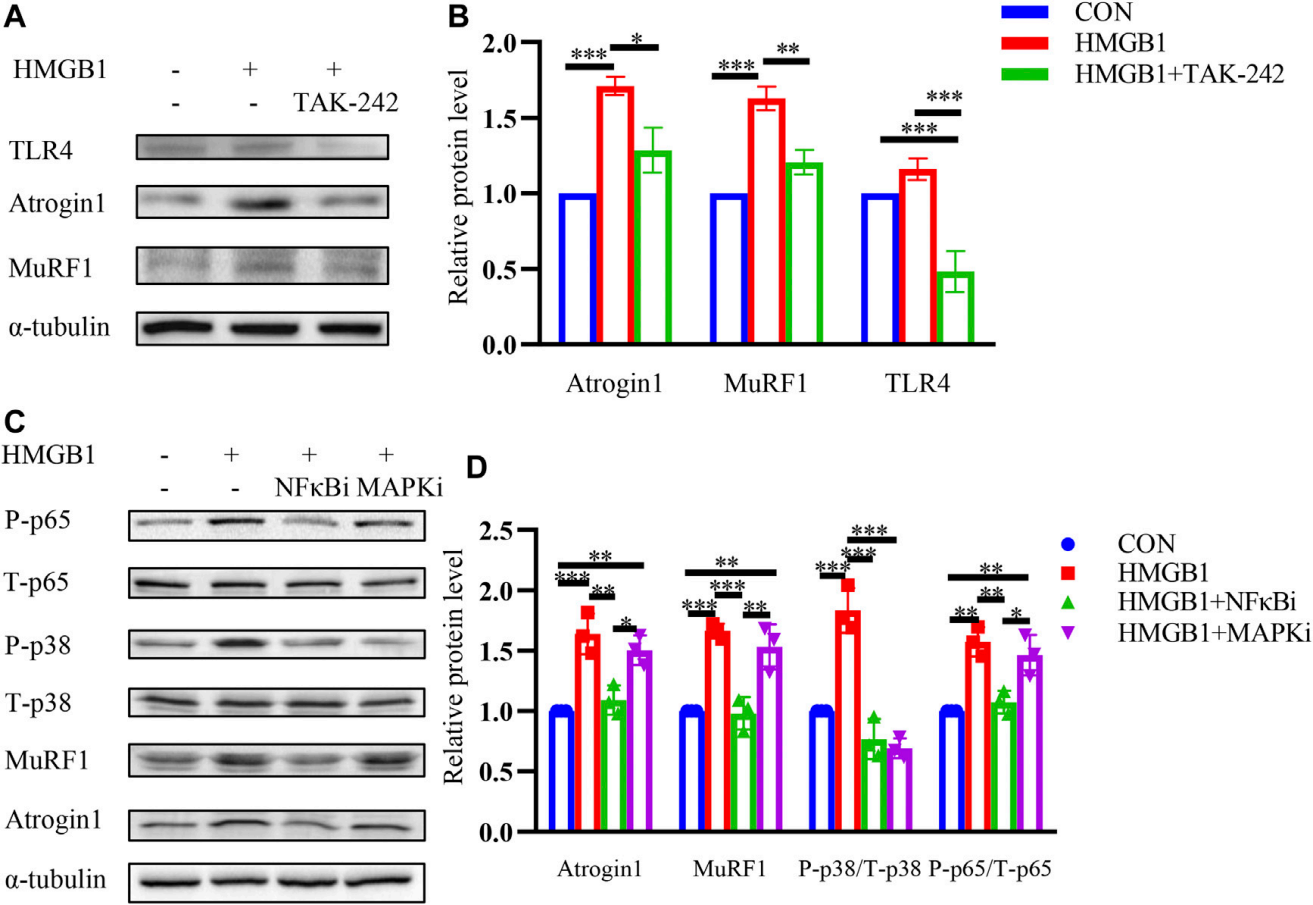
HMGB1 induces muscle wasting through TLR4/NF-κB signaling pathway. **(A)** C2C12 myotubes were pretreated with inhibitor for TLR4 (TAK-242) followed by incubation with HMGB1. The expression of Atrogin1 and MuRF1 was determined by western blot. **(B)** Densitometric quantification of the Atrogin1 and MuRF1 signal relative to the α-tubulin signal. **(C)** C2C12 myotubes were pretreated with inhibitors for NF-κB and MAPK pathways followed by incubation with HMGB1. The expression of Atrogin1, MuRF1, phosphorylated p65 and phosphorylated p38 was determined by western blot. **(D)** Densitometric quantification of the Atrogin1 and MuRF1 signal relative to the α-tubulin signal, phosphorylated p65 relative to the total p65 signal and phosphorylated p38 signal relative to the total p38 signal.

To further explore the signaling pathway influenced by HMGB1, we used TLR4 inhibitor TAK-242 (10 μM) pretreated with myotubes followed by HMGB1. As shown in Figures 4A,B, muscle wasting caused by HMGB1 could be partly reversed by TAK-242, suggesting that HMGB1 may induce muscle wasting through TLR4. NF-κB signaling pathway and MAPK signaling pathway are two known TLR4 downstream pathways and two important pathways in cancer cachexia. To further explore the signaling pathways, we used the NF-κB signaling pathway inhibitor BAY 11-7082 (10 μM) and MAPK signaling pathway inhibitor PD169316 (10 μM) to find the downstream signaling pathway. NF-κB inhibitor could inhibit the upregulation of the atrophy-related proteins Atrogin1 and MuRF1 (Figures 4C,D). MAPK signaling pathway inhibitor could slightly inhibit the upregulation of the atrophy-related proteins Atrogin1. We could infer that NF-κB may be the dominant signaling pathway in HMGB1 mediated muscle wasting. We also found that phosphorylated p65 (p-p65) and p38 (p-p38) upregulated in HMGB1 treated myotubes. Moreover, NF-κB inhibitor could reverse the activation of MAPK pathway, indicating the major role of NF-κB pathway. These results revealed that HMGB1 induced muscle wasting mainly through TLR4/NF-κB signaling pathway.

### HMGB1 Inhibitor Glycyrrhizin Reverses Proteolysis of C2C12 Myotubes Induced by HMGB1 and CT26-Derived Exosomes

**FIGURE 5:**
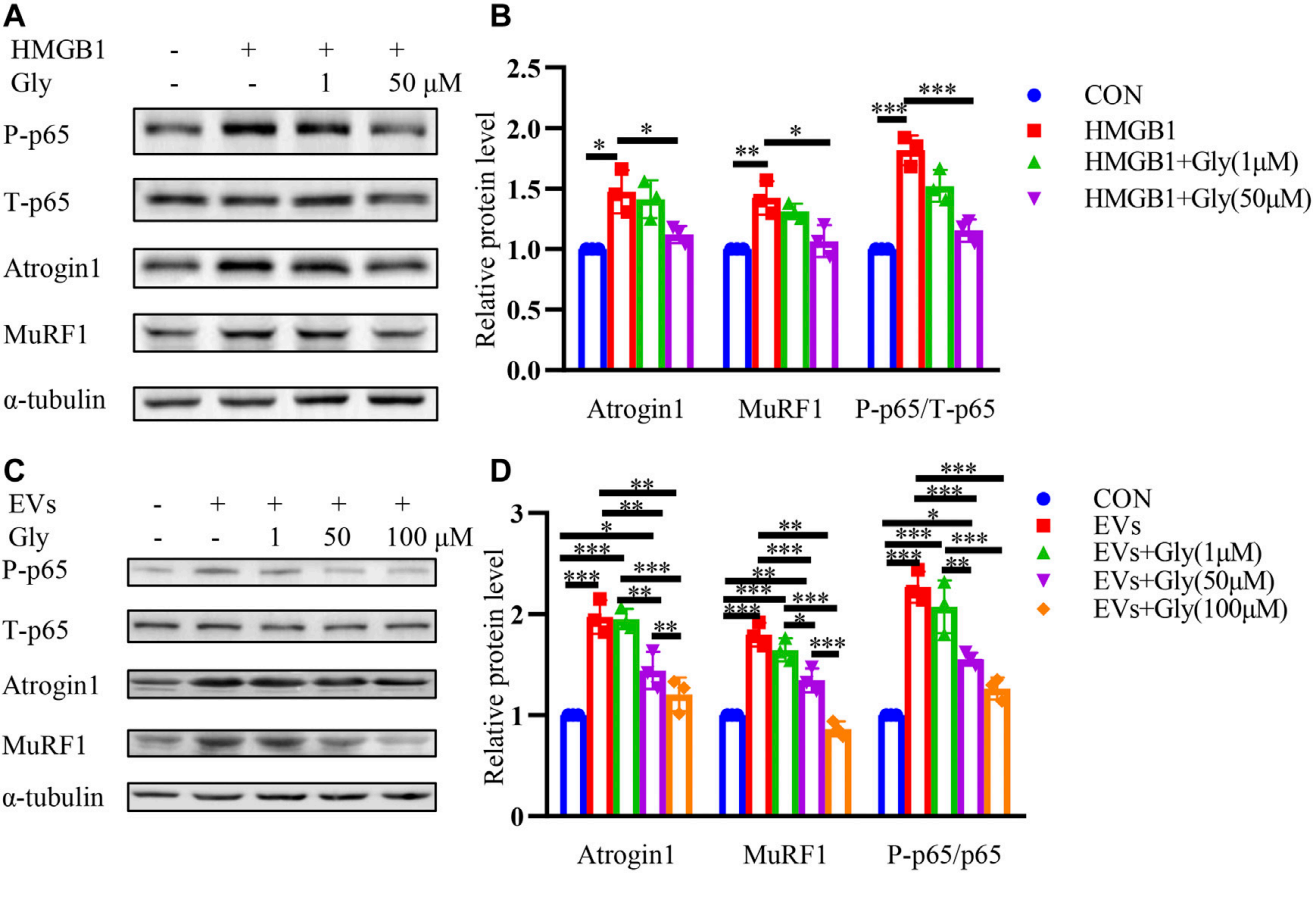
HMGB1 inhibitor glycyrrhizin (Gly) reverses proteolysis of C2C12 myotubes induced by HMGB1 and CT26-derived exosomes. **(A)** Total extracts of C2C12 myotubes treated with HMGB1, HMGB1 and 1 μM Gly, HMGB1 and 50 μM Gly were used for western blotting analysis with antibodies against with P-P65, P65, Atrogin1, MuRF1 and α-tubulin. **(B)** Densitometric quantification of the P-P65 signal relative to total P65 signal and the Atrogin1 and MuRF1 signal relative to the α-tubulin signal. **(C)** Total extracts of C2C12 myotubes treated with EVs, EVs and 1 μM Gly, EVs and 50 μM Gly, EVs and 100 μM Gly were used for western blotting analysis with antibodies against with P-p65, P65, Atrogin1, MuRF1 and α-tubulin. **(D)** Densitometric quantification of the P-p65 signal relative to total P65 signal and the Atrogin1 and MuRF1 signal relative to the α-tubulin signal.

Glycyrrhizin is reported to bind with HMGB1 directly and inhibit HMGB1 (Li et al., 2017; Wu et al., 2018; Wang et al., 2021). To further explore whether the HMGB1 inhibitor glycyrrhizin could reverse the muscle atrophy, we investigated its effects on HMGB1 induced muscle atrophy. HMGB1-induced elevation of Atrogin1 and MuRF1 were inhibited by glycyrrhizin and the decrease of phosphorylated p65 was also observed (Figures 5A,B). Furthermore, EVs induced muscle wasting could also be partially relieved by glycyrrhizin (Figures 5C,D).

### Down Regulating HMGB1 Alleviates Muscle Wasting

**FIGURE 6:**
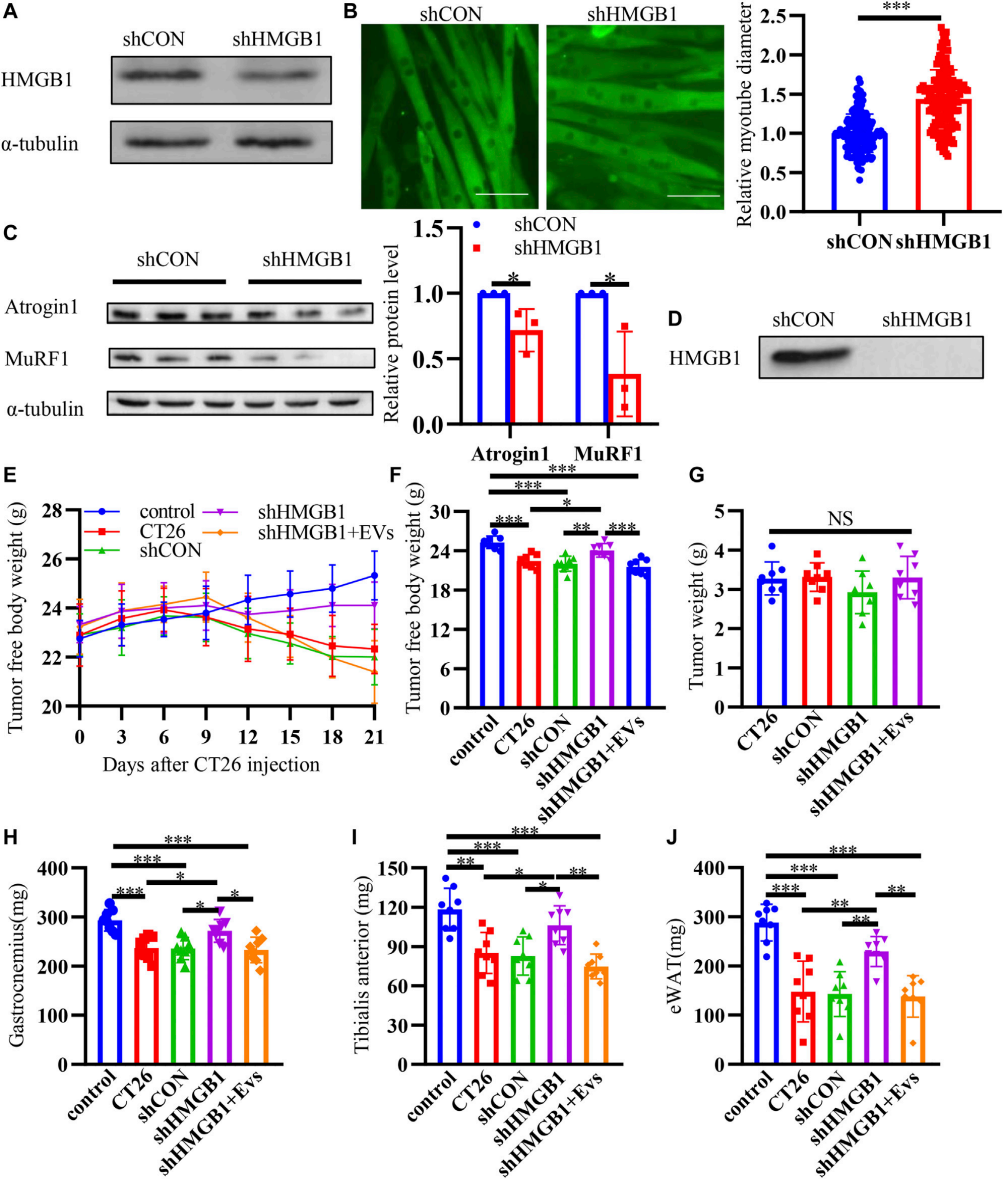
Down regulating HMGB1 alleviates muscle wasting. **(A)** Decreased expression of HMGB1 was determined by western blot after knocking down of HMGB1 in CT26 cell lines. **(B)** Representative immunofluorescence images for C2C12 myotubes treated with normal CT26-EVs and HMGB1-lowexpressed CT26-EVs. The relative fiber widths of C2C12 myotubes treated with normal CT26-EVs and HMGB1-lowexpressed CT26-EVs. **(C)** Total extracts of C2C12 myotubes treated with normal CT26-EVs and HMGB1-lowexpressed CT26-EVs were used for western blotting analysis with antibodies against with Atrogin1, MuRF1 and α-tubulin. Densitometric quantification of the Atrogin1 and MuRF1 signal relative to the α-tubulin signal. **(D)** HMGB1-lowexpressed CT26-EVs contained low level of HMGB1 compared with normal CT26-EVs. **(E)** Tumor-free body weight growth curve of BALB/c mice. **(F)** Tumor-free body weight of BALB/c mice at 21 days of tumor implantation. Tumor weight **(G)**, bilateral gastrocnemius weight **(H)**, bilateral tibialis anterior weight **(I)** and bilateral epididymal weight (eWAT) **(J)** of BALB/c mice at 21 days of tumor implantation.

In order to further prove the effect of HMGB1 in cancer cachexia, we used lentivirus to knock down the expression of HMGB1 in CT26 cells for cell and animal experiment. *In vitro*, we found that HMGB1 was significantly down-regulated after transduction (Figure 6A). Then, we collected EVs from transduced cells. Immunofluorescence staining assay revealed that myotube diameter became longer after HMGB1 knocking down (Figure 6B). And we also detected decreased MuRF1 and Atrogin1 in myotubes incubated with HMGB1-lowexpressed CT26-EVs (Figure 6C). Furthermore, we detected low level of HMGB1 in HMGB1-lowexpressed CT26-EVs (Figure 6D). *In vivo*, we found that the shHMGB1 group mice had significantly higher tumor-free body weight compared with other tumor-bearing mice at the end of the experiment (Figures 6E,F). There were no significant differences in tumor weights between groups (Figure 6G). The muscle loss of gastrocnemius (Figure 6H) and tibial anterior (Figure 6I) were reduced and the epididymal fat increased (Figure 6J). While, EVs injection abrogated the effects. We could infer that tumoral HMGB1 is one of the cachexic factors and exosomes containing HMGB1 might be one of the mediators between tumor and muscle.

### HMGB1 Inhibitor Glycyrrhizin Alleviates Cancer Cachexia in CT26 Tumor-Bearing Mice

**FIGURE 7:**
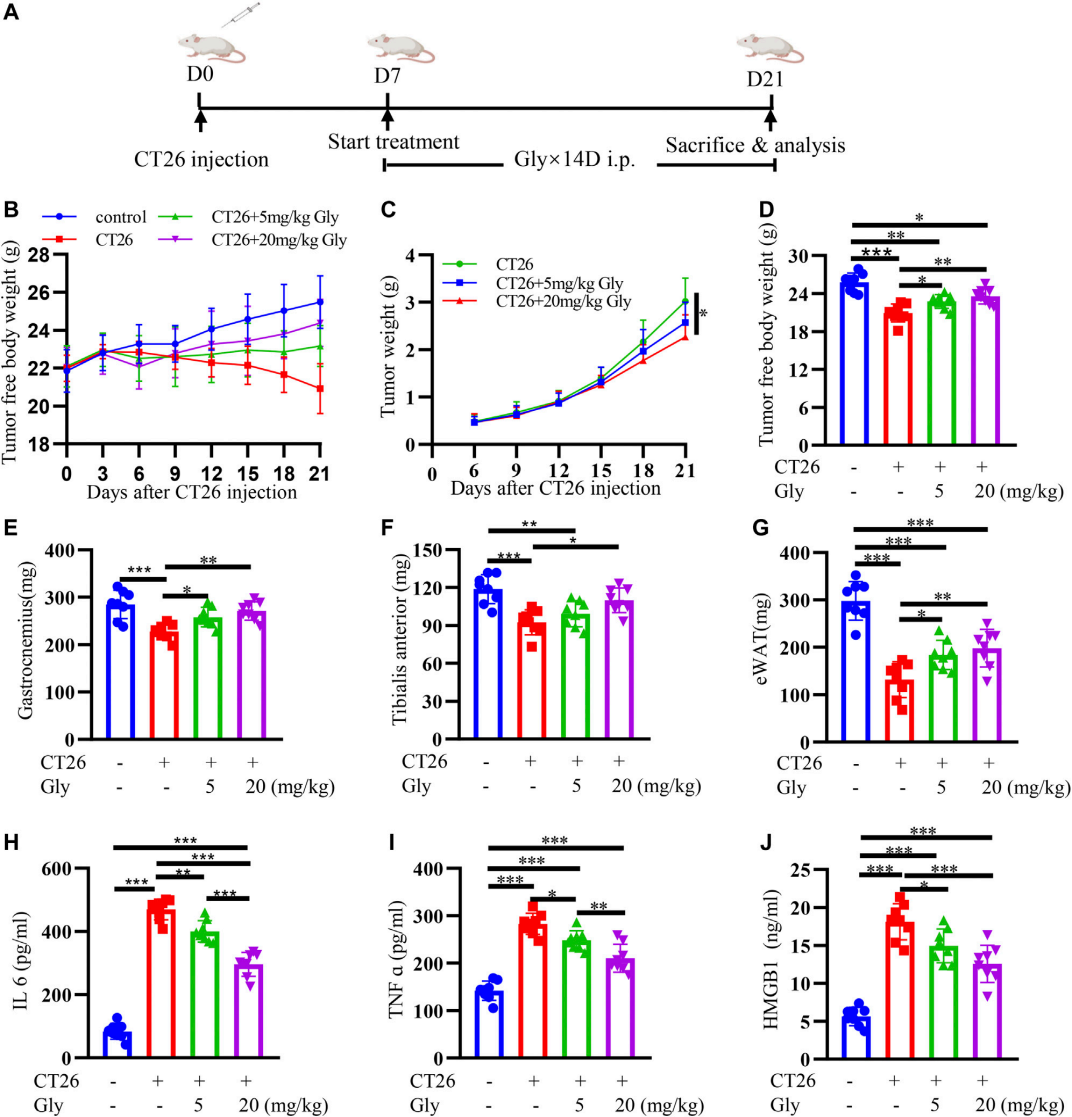
HMGB1 inhibitor glycyrrhizin alleviates cancer cachexia in CT26 tumor-bearing mice. **(A)** Schematic presentation of the animal experiment protocol **(B)** Tumor-free body weight growth curve of BALB/c mice. **(C)** The tumor weight curve of BALB/c mice. The lean body weight **(D)**, bilateral gastrocnemius weight **(E)**, bilateral tibialis anterior weight **(F)** and bilateral epididymal weight **(G)** of BALB/c mice at 21 days of tumor implantation. The serum IL6 **(H)**, TNF α **(I)**, HMGB1 **(J)** in BALB/c mice.

In order to promote the clinical application of HMGB1, we used a commercial HMGB1 inhibitor glycyrrhizin to conduct murine experiment. The mice experiment was conducted according to the protocol in Figure 7A. On the seventh day after tumor cell injection, when tumors were palpable, we started intraperitoneal administration. CT26 tumor-bearing mice showed darker fur, slower activity and weight loss compared with normal mice at about 10 days after tumor inoculation. Weights of mice were recorded every 3 days (Figure 7B). The tumor weights of CT26 + 20 mg/kg glycyrrhizin group were significantly lower than that of CT26 group (Figure 7C). Cachexia mice showed significantly decreased lean body weight (Figure 7D), gastrocnemius (Figure 7E), tibialis anterior (Figure 7F) and epididymal fat (Figure 7G) at day 21 when killed. The decrease of the lean body mass was relieved after the administration of glycyrrhizin. Glycyrrhizin prevented decreased weight of the gastrocnemius, tibialis anterior, and epididymal fat in tumor-bearing mice. What’s more, we found decreased inflammatory factors IL-6 (Figure 7H), TNF α (Figure 7I) and HMGB1 (Figure 7J) in glycyrrhizin group. We could infer that glycyrrhizin alleviated cachexia in CT26 tumor-bearing mice by alleviating weight loss, increasing muscle weight, reserving adipose tissues, and relieving systematic inflammation.

### HMGB1 Inhibitor Glycyrrhizin Alleviates Muscle Wasting Through NF-κB Signaling Pathway in CT26 Tumor-Bearing Mice

**FIGURE 8:**
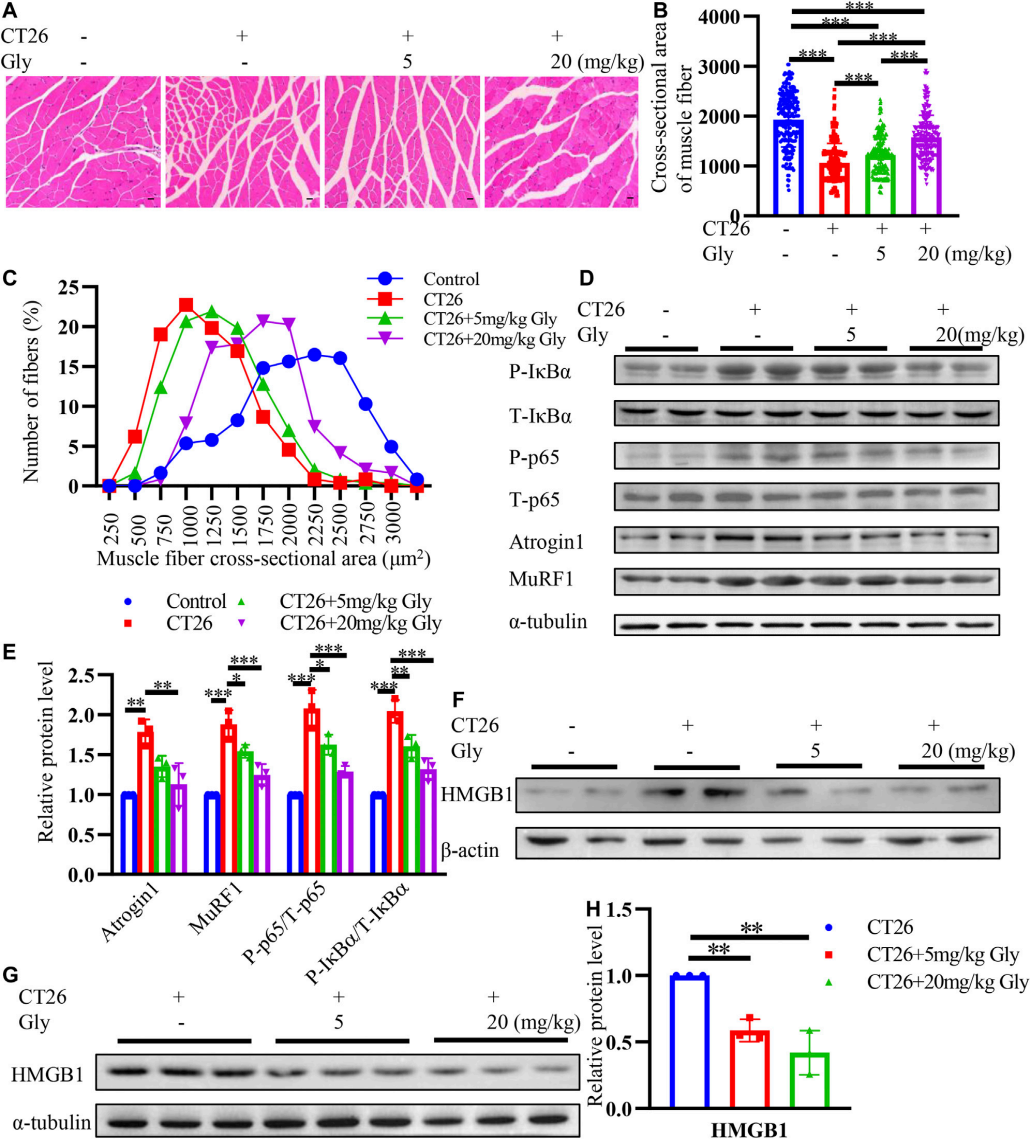
HMGB1 inhibitor glycyrrhizin alleviates muscle wasting through NF-κB signaling pathway in CT26 tumor-bearing mice. **(A)** Hematoxylin and eosin (HE) staining of gastrocnemius muscle. Scale bar = 100 μm **(B)** Cross-sectional area analysis of gastrocnemius muscle. **(C)** Distribution of gastrocnemius cross-sectional area. **(D)** Total extracts of gastrocnemius muscle were used for western blotting analysis with antibodies against with P-p65, P65, P-IκB, IκB, Atrogin1, MuRF1 and α-tubulin. **(E)** Densitometric quantification of the P-P65 signal relative to total P65 signal, P-IκB signal relative to total IκB signal, the Atrogin1 and MuRF1 signal relative to the α-tubulin signal. **(F)** HMGB1 levels in mice serum exosomes. **(G)** Total extracts of tumor tissues were used for western blotting analysis with antibodies against with HMGB1. **(H)** Densitometric quantification of the HMGB1 signal relative to the α-tubulin signal.

**FIGURE 9:**
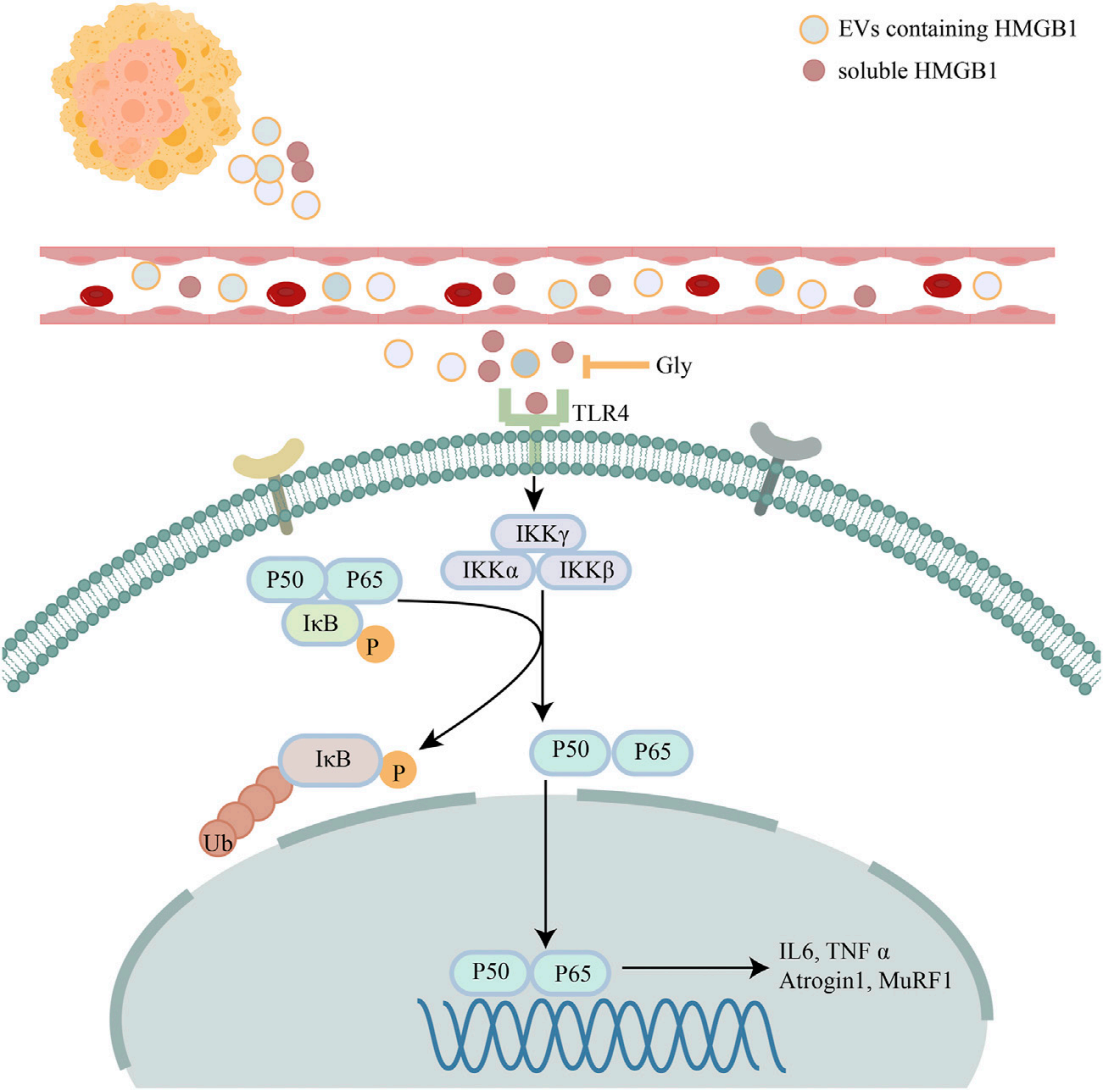
Schematic diagram describing the role of HMGB1. Schematic diagram describing the mechanism of HMGB1 mediated muscle wasting though TLR4/NF-κB signaling pathway.

To further explore the effect of glycyrrhizin on cachexia mice, we conducted HE staining on cross-sectional area of gastrocnemius (Figure 8A). We found that glycyrrhizin could alleviate the shrinkage of muscle fibers (Figures 8B,C). Western blotting analysis revealed that glycyrrhizin inhibited the muscle atrophy markers Atrogin1 and MuRF1 as well as the NF-κB signaling pathway (Figures 8D,E). Furthermore, we detected low level of HMGB1 in mice serum EVs (Figure 8F) and tumor tissues (Figures 8G,H) treated with glycyrrhizin. We further confirmed the HMGB1 inhibiting role of glycyrrhizin in mice model and verified that the down regulating of the NF-κB signaling pathway and muscle atrophy markers in muscle tissues. The schematic diagram describing the role of HMGB1 was represented in Figure 9.

## Discussion

Cancer cachexia is a metabolic systemic disease depicted by decreased body weight, systemic inflammation and muscle wasting with or without fat loss. Maintaining skeletal muscle mass is of great significance for improving the quality of life and prolonging survival time. However, so far in clinical practice, there is no effective way to relieve muscle loss. Exploration of potential biomarkers and effective anti-cachexia drugs is imperative.

In a systematic literature review of 18 studies involving 11 cancers, high expression of HMGB1 was associated with poor prognosis (Wu et al., 2016). Elevated HMGB1 is associated with malignant phenotypes, including tumor invasion and metastasis (Luo et al., 2010; Liu et al., 2011; Luo et al., 2013). Serum HMGB1 is higher in colorectal carcinoma and HMGB1 is a valuable diagnostic biomarker (Lee et al., 2012). In patients with colorectal cancer (CRC), high serum HMGB1 was positively correlated with lymph node metastasis (Zhang et al., 2019). Knockdown of endogenous HMGB1 with short hairpin RNA (shRNA) can inhibit the proliferation of cancer cells by reducing Bcl-2 and activating Bax (Wang et al., 2016). Exosomes containing HMGB1 have been reported in gastric cancer and esophageal cancer to facilitate tumor malignancy (Zhang et al., 2018; Li et al., 2019a; Shi et al., 2020). However, there are relatively few reports on the relationship between HMGB1 and cachexia. In this study, we found that tumor-derived exosome was a cachexia factor *in vitro* and CT26-EVs contained a high level of HMGB1. Exosomes take a pivotal role in signal transduction between tissues. Also, we demonstrated that serum HMGB1 was elevated in patients with cachexia. At the cellular level, HMGB1 could induce myotube atrophy. While, in our study, we could hardly find HMGB1 in Lewis lung cancer-derived exosomes, another classic cachexia model (data not shown), which may indicate that different tumor types have different cachexins. To further confirm the role of HMGB1 in muscle wasting, we knocked down HMGB1 in CT26 cells and HMGB1-lowexpressed CT26 tumor-bearing mice showed alleviated cachexia symptoms.

Glycyrrhizin is the most important active ingredient extracted from licorice root. It has a broad spectrum of anti-cancer and anti-inflammatory properties. It can also reduce the level of alanine aminotransferase and protect the liver (Li et al., 2014; Li et al., 2019b). It can directly bind to HMGB1 and inhibit the binding of HMGB1 to other receptors (Mollica et al., 2007). Thereby inhibiting the downstream activation of RAGE and TLRs. Glycyrrhizin acid can induce the apoptosis of colon cancer tumor cells SW48 in a dose-dependent manner without affecting normal colonic epithelial cell (Zhang et al., 2020). In our study, glycyrrhizin could effectively alleviate the muscle atrophy induced by recombinant HMGB1 and CT26-EVs. At the same time, HMGB1 led to the activation of the TLR4/NF-κB signaling pathway, thereby activating the ubiquitin proteasome system, and glycyrrhizin could effectively inhibit the activation of this pathway. At the animal level, glycyrrhizin alleviated cachexia in CT26 tumor-bearing mice with increased muscle mass and decreased inflammation factors. Cachexia relieving seemed to have nothing to do with the inhibition of tumor growth. Because in the low-dose group with no significant difference in tumor weight, glycyrrhizin could alleviate cachexia to a certain extent. Systemic inflammation is one of the main characteristics of cancer cachexia. Studies have shown that cachexia patients have higher serum TNF-α and IL-6 levels (Riccardi et al., 2020; Webster et al., 2020). Elevated TNF-α and IL-6 will activate the ubiquitin proteasome system through the NF-κB pathway. At the same time, the activation of NF-κB will also increase the transcription of TNF-α and IL-6, further aggravating inflammatory response. Glycyrrhizin has also been reported to attenuate carcinogenesis in an azoxymethane/dextran sodium sulfate-induced colorectal cancer model (Wang et al., 2021). In that model, glycyrrhizin could lower the levels of TNF α and IL-6, which is consistent with our experiment.

In conclusion, we found that exosomes containing HMGB1 could induce muscle wasting and serum HMGB1 was elevated in cachexia patients. Glycyrrhizin, HMGB1 inhibitor, may be a potent drug in cancer cachexia.

## Data Availability

The original contributions presented in the study are included in the article/Supplementary Material, further inquiries can be directed to the corresponding author.
