# Supplementary material for: Pharmacological Inhibition of HMGB1 Prevents Muscle Wasting
Source: Front Pharmacol. 2021 Nov 18;12:731386. doi: 10.3389/fphar.2021.731386 (PMC8637759; doi:10.3389/fphar.2021.731386)

Supplymentary figure. Replicates of western blotting.

Figure 1C

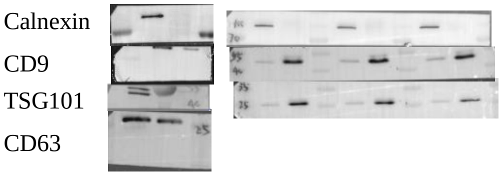

Figure 2A

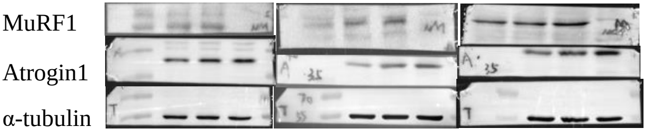

Figure 2F

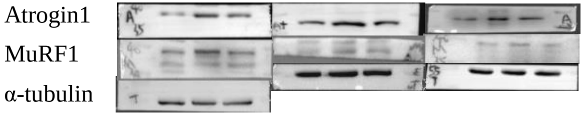

Figure 3B

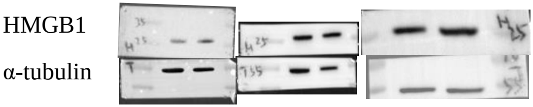

Figure 3D

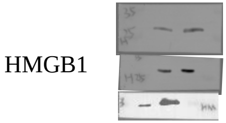

Figure 3E

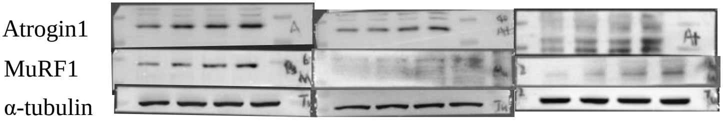

Figure 4A

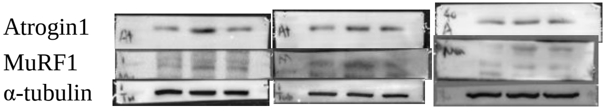

Figure 4C

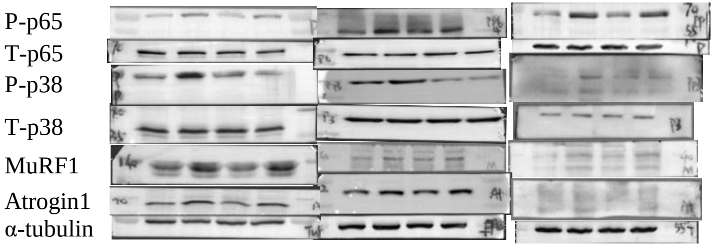

Figure 5A

P-p65
T-p65
Atrogin1
MuRF1
α-tubulin

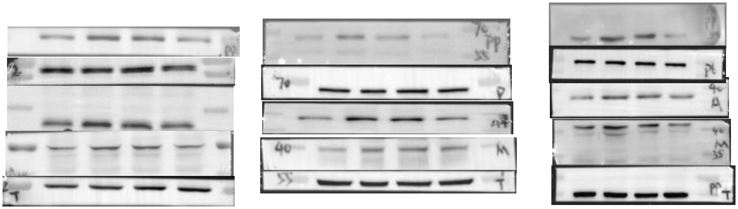

Figure 5C

P-p65
T-p65
Atrogin1
MuRF1
α-tubulin

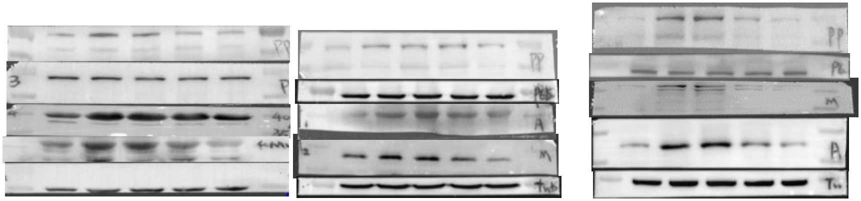

Figure 6A

HMGB1
α-tubulin

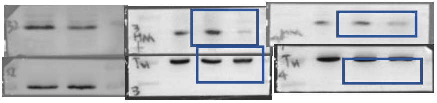

Figure 6D

HMGB1

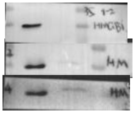

Figure 6C

Atrogin1
MuRF1
α-tubulin

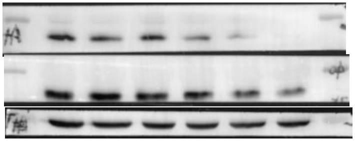

Figure 8F

HMGB1
β-actin
HMGB1

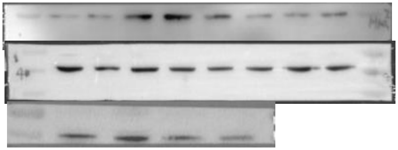

Figure 8D

P-IκBα
T-IκBα
P-p65
T-p65
Atrogin1
MuRF1
α-tubulin

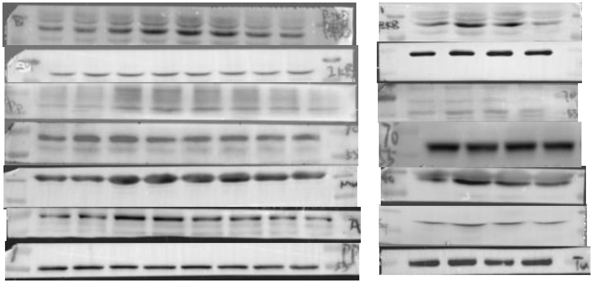

Figure 8G

HMGB1
α-tubulin

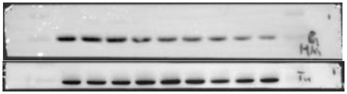

Supplement: Supplementary file 1 [file DataSheet1.pdf]
